# Long-Term Cost and Health Impact of a Digital Obesity Intervention in Germany: Economic Modeling Study

**DOI:** 10.2196/88576

**Published:** 2026-08-24

**Authors:** Evelyn Walter, Marisa Kaup, Annina Eva Althaus, Leonard Fehring

**Affiliations:** 1 Institute for Pharmaeconomic Research Vienna, Vienna Austria; 2 Oviva AG Berlin Germany; 3 Ovivamed, Oviva AG Zürich Switzerland; 4 Institute of Social Medicine and Epidemiology Brandenburg Medical School Theodor Fontane Brandenburg an der Havel Germany; 5 Health Care Informatics, Faculty of Health School of Medicine, Witten/Herdecke University Witten Germany; 6 Medizinische Klinik 2 Helios University Hospital Wuppertal Wuppertal Germany

**Keywords:** cost-effectiveness, cost-utility, DHA, DiGA, digital health apps, digital health applications, obesity, overweight

## Abstract

**Background:**

Obesity imposes a substantial economic burden, accounting for an estimated 10% of total health care expenditures in Germany. Digital health apps (DHAs) have demonstrated effectiveness in supporting weight management among individuals with obesity; however, evidence regarding their long-term economic impact remains limited.

**Objective:**

This study aimed to evaluate the long-term cost-effectiveness of the Oviva Direkt app for obesity treatment in the German health care context.

**Methods:**

We developed a cohort-based Markov model, informed by the Core Obesity Model, to evaluate the cost-effectiveness of a DHA for weight management vs care as usual over a 10-year time horizon from a societal perspective. The model simulates disease progression using 6-month and annual cycles after an initial 6-week phase and includes health states for key obesity-related comorbidities such as type 2 diabetes (T2D), acute coronary syndrome, stroke, cancer, and obstructive sleep apnea. Patients enter the model at age 46 years with a BMI of 30 to 45 kg/m^2^, based on trial data. The analysis considered direct and indirect costs, life-years, and quality-adjusted life-years (QALYs). The primary outcome was the incremental cost-effectiveness ratio, complemented by net monetary benefit analysis. Weight trajectories were extrapolated from trial results using 3 scenarios (base-case decay model, weight maintenance, and full regain). Sensitivity analyses were conducted to assess uncertainty.

**Results:**

Under base-case assumptions, the DHA dominated care as usual, yielding cost savings of €3511.85 (€1=US $1.127 as of May 2025) and a QALY gain of 0.0683 (approximately 3.6 weeks in perfect health). Direct medical costs were reduced by nearly €520. The prevalence of T2D was 1.6 percentage points lower, reducing time lived with diabetes by 8 months. Scenario analyses confirmed consistent cost-effectiveness.

**Conclusions:**

DHAs for weight management are cost-effective and clinically beneficial for individuals with obesity in Germany. These results add to the growing evidence for digital health solutions, aligning with findings from applications for other conditions such as depression and multiple sclerosis.

## Introduction

The prevalence of overweight and obesity has increased dramatically across Europe and worldwide, representing a major public health challenge. According to the Robert Koch Institute, 46.6% of women and 60.5% of men in Germany are currently overweight, with 19% of adults classified as obese [1]. Over recent decades, obesity rates have risen steadily across all age groups, driving a growing burden on health care systems. Overweight and obesity are associated with a wide range of comorbidities, including hypertension, dyslipidemia, coronary heart disease, heart failure, type 2 diabetes (T2D), nonalcoholic fatty liver disease, obstructive sleep apnea, and depression or anxiety, that together contribute to increased morbidity, reduced quality of life, and escalating health care costs [2]. Despite the clinical need, access to treatment remains limited because of the scarcity of effective therapeutic options and inadequate reimbursement. Consequently, only a small proportion of people with obesity in Germany currently receive care [3].

Beyond clinical implications, the economic impact of obesity is substantial. A systematic review across multiple countries found that annual health care costs are on average 12% higher for overweight and 36% higher for individuals with obesity compared with those with normal weight [4]. These costs include both direct medical expenses and indirect costs such as absenteeism and early retirement. Excluding indirect costs leads to a significant underestimation of the true economic burden of obesity, as indirect costs often exceed direct expenditures [4]. A recent Finnish cohort study reported 31% to 95% higher annual total direct costs among individuals with overweight and obesity compared with those with normal weight, largely driven by reduced work capacity [5]. This finding aligns with previous research highlighting that indirect costs dominate the overall economic impact of obesity [6,7].

Over the past decade, digital transformation has reshaped health care delivery. The rapid uptake of smartphones, tablets, and wearable devices has enabled the use of mobile health (mHealth) apps to support the prevention and management of chronic diseases. These digital tools can empower individuals by fostering health literacy, promoting behavior change, and enhancing adherence to therapy, thereby improving engagement and long-term outcomes [8,9]. Mobile apps are increasingly used in obesity care to deliver scalable, personalized interventions that support self-monitoring, feedback, and remote professional guidance, factors known to enhance self-efficacy and facilitate behavior change [10,11], and offer an innovative approach to obesity management.

Several meta-analyses of randomized controlled trials have shown that mobile app–based interventions can lead to modest but statistically significant weight loss and behavioral improvements in adults with overweight or obesity. A 2025 meta-analysis of 11 RCTs (n=1717) reported a mean weight reduction of −0.76 kg (95% CI −1.42 to −0.10; *P*=.02) and a decrease in body fat percentage of −0.46% (95% CI −0.71 to −0.20) after 4 to 6 months of app use without additional support [12]. Another review found a peak weight loss of −2.18 kg at 3 months, with a maintained effect of −1.63 kg after 12 months [13]. Apps incorporating behavior change techniques, such as goal setting, self-monitoring, personalized feedback, and reminders, tend to be more effective [13]. However, evidence on the long-term sustainability and economic value of such interventions remains limited. To date, no study has conducted a full cost-utility analysis of a digital intervention for weight management using quality-adjusted life-years (QALYs) as the outcome.

Germany has pioneered the integration of evidence-based digital tools into its statutory health care system through the Digital Health Applications (DiGA) framework, which allows certified digital health interventions to be prescribed and reimbursed as part of standard care. This framework provides a unique opportunity to generate real-world evidence on the clinical and economic value of digital therapies. Yet, the cost-effectiveness of digital health apps (DHAs) for obesity management remains uncertain, and data to guide reimbursement and policy decisions are scarce.

A recent randomized controlled trial (registration number DRKS00033045; registration date November 14, 2023) investigated a widely prescribed, reimbursable DHA for weight management in Germany. The app delivers personalized dietary counseling, behavioral strategies, and regular remote support from health care professionals. After 6 months, participants in the intervention group achieved a mean weight loss of 5.29% (95% CI −6.73% to −3.86%; *P*<.001) compared with 1.76% (95% CI −3.10% to −0.42%; *P*=.01) in the control group [14].

The Oviva Direkt app, which combines structured nutritional counseling, behavior change techniques, and digital coaching to promote sustainable weight loss and improved health outcomes, is currently the most frequently prescribed DHA in Germany. It thus provides a particularly relevant case in which to examine the economic and clinical value of app-based obesity interventions. Building on evidence from the randomized controlled trial, we developed a cost-utility model assessing the long-term cost-effectiveness of this digital approach in the German health care context. The model adopts a societal perspective, capturing both direct medical and indirect productivity costs.

Evidence on the economic value of digital weight-management interventions is still limited. Only a few studies have modeled long-term cost-effectiveness, and recent reviews highlight a lack of robust cost-utility analyses in this field [15-17].

To our knowledge, this study is the first cost-utility analysis of a DiGA-certified DHA for obesity management in the German statutory health care context. By integrating clinical and economic data, it provides novel insights into the cost-effectiveness of scalable, app-based weight-management programs. Given the global burden of obesity and the increasing role of digital health tools, scalable and economically sustainable interventions are urgently needed. Our findings aim to inform payers, providers, and policymakers on the feasibility and value of DHAs for obesity care while highlighting priorities for future research on long-term adherence, sustained outcomes, and implementation across diverse health care systems.

## Methods

### Ethical Considerations

The present study was based on secondary analyses of data from a randomized controlled trial evaluating the Oviva Direkt DHA in adults with obesity in Germany (DRKS00033045). The underlying trial was approved by the Ethics Committee of the Berlin Medical Association (Eth-57/23), and all participants provided written informed consent prior to participation. No additional participant contact or data collection was conducted for the present economic evaluation.

### Model Design

Over the first 6 months, the model was informed by individual patient data from a randomized controlled trial evaluating the Oviva Direkt app in adults with obesity in Germany (DRKS00033045) [14]. Adults aged 18 to 75 years with physician-diagnosed obesity (BMI 30 to 45 kg/m^2^), access to a compatible smartphone, and no recent or planned antiobesity pharmacotherapy were randomized to receive either the DHA or care as usual. Care as usual consisted of routine lifestyle counseling and weight-management advice provided at the discretion of the treating physician, without structured digital coaching. The primary clinical end point was percentage weight change at 6 months. The body weight and BMI data used to parameterize the short-term treatment effect are provided in Multimedia Appendix 1 [14].

The economic model used the observed 6-month weight-change data, stratified by treatment arm, to derive BMI distributions at model entry and to parameterize weight-change trajectories over the 10-year horizon. The Markov model was adapted from the published Core Obesity Model (COM) by Lopes et al [18] and applies a decision-analytic framework to project long-term clinical and economic outcomes beyond the randomized controlled trial. Clinical, epidemiological, utility, and cost inputs were identified from published literature and other publicly available sources up to June 2025. Model parameters not informed by the randomized controlled trial were derived from the best available evidence and adapted to the German health care context where appropriate.

The model uses 6-week cycles during the 24-week trial period, corresponding to the visit schedule of the underlying randomized controlled trial, followed by a 6-month cycle for the remainder of the first year and annual cycles thereafter. Annual cycles were considered appropriate beyond the first year, as changes in BMI and obesity-related complications occur more gradually over longer time horizons. Transition probabilities were converted between cycle lengths using standard exponential rate transformations. Probabilities reported for a given time interval were first converted into continuous rates 
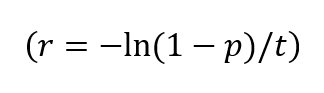
 and subsequently transformed to the desired cycle length 
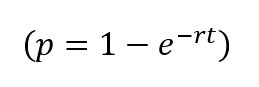
. A half-cycle correction was applied to incident clinical events.

Weight trajectories determine transitions between BMI categories and were linked to changes in the prevalence (baseline) and incidence (follow-up cycles) of obesity-related comorbidities. BMI-specific risks for incident comorbidities were incorporated using Cox proportional hazard ratios (HRs) from the COM, stratified by BMI, age, and sex. The following comorbidities were included: (1) prediabetes, (2) T2D, (3) acute coronary syndrome (ACS: angina and myocardial infarction [MI]), (4) stroke, (5) obstructive sleep apnea (OSA), (6) obesity-related cancers (colorectal, postmenopausal breast, and endometrial), and (7) all-cause mortality.

**Figure 1:**
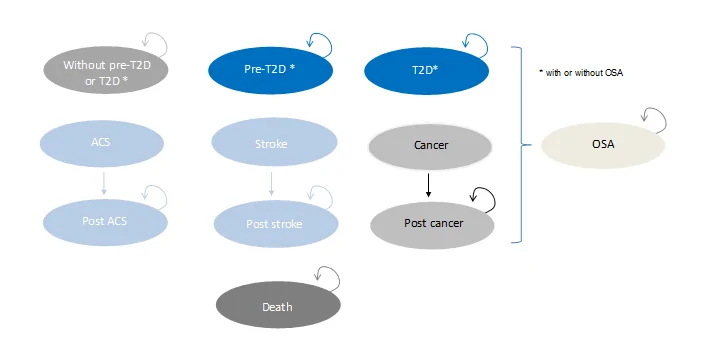
Structure of the Markov model. The model applies 6-week cycles during the trial period, followed by a 6-month cycle and then annual cycles over the 10-year time horizon. The following comorbidities were included: (1) prediabetes (pre–type 2 diabetes [T2D]), (2) T2D, (3) acute coronary syndrome (ACS: angina and MI), (4) stroke, (5) obstructive sleep apnea (OSA), (6) cancer (colorectal, postmenopausal breast, and endometrial), and (7) all-cause mortality.

At model entry, individuals were distributed across BMI categories of 30.0-34.9 kg/m^2^, 35.0-39.9 kg/m^2^, and 40.0-44.9 kg/m^2^, reflecting the trial population. Additional BMI categories (18.5-24.9 kg/m^2^ and 25.0-29.9 kg/m^2^) were included to capture weight reduction over time. Individuals were also stratified by glycemic state (normoglycemia, prediabetes, and T2D). In each cycle, individuals could develop 1 or more comorbidities based on BMI-, age-, and sex-specific incidence rates; all health states included an associated mortality risk (Figure 1).

To assess the impact of the DHA compared to care as usual, the model evaluated 2 strategies:

Intervention: participants in the intervention arm received a DHA for weight management for a duration of 6 months. The app-based, multimodal program is aligned with the German guidelines for the prevention and treatment of obesity [19] and includes digital coaching, nutritional counseling, and behavioral support.

Control: participants in the control group received care as usual, typically consisting of general lifestyle advice or counseling provided at the discretion of their general practitioner, without structured digital support, as implemented in the underlying randomized controlled trial.

### Data Analysis

The model reports cumulative direct and indirect costs, clinical outcomes expressed as QALYs gained, life-years gained, and patient-years lived with comorbidities. Results were evaluated from both the payer and societal perspectives.

The primary analysis was based on the base-case scenario. In addition, optimistic and pessimistic scenarios were evaluated to represent a plausible range of long-term weight trajectories. The definitions of these scenarios are provided in the Weight Development section.

The primary outcome of the economic evaluation is the incremental cost-effectiveness ratio (ICER), which represents a combined measure showing the additional costs per QALY gained. The ICER is calculated by dividing the difference in total costs between the intervention and control groups by the difference in their health outcomes (eg, QALYs gained), as defined by Drummond et al [20]. Formally, the ICER is expressed as follows, where C₁ and E₁ denote the total costs and effects (eg, QALYs) in the intervention group, and C₂ and E₂ denote those in the control group. The ICER thus reflects the additional cost required to achieve 1 additional unit of health benefit, typically expressed as cost per QALY gained [20]:

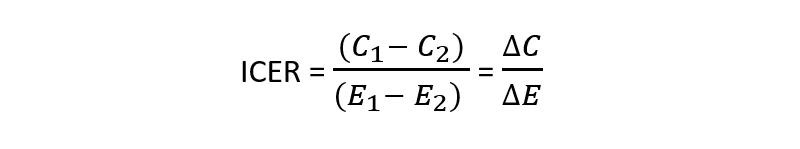



Germany does not define an explicit willingness-to-pay (WTP) threshold for cost-effectiveness analyses. However, in international practice, interventions are often considered cost-effective if the incremental cost per QALY gained falls below the country’s gross domestic product per capita—approximately €51,833 (2024; €1=US $1.127 as of May 2025) in Germany. Based on this benchmark, the net monetary benefit (NMB) framework is used to quantify the overall value of an intervention in monetary terms. The NMB incorporates both costs and effects, allowing for a direct comparison of the economic value of the app-based weight-management intervention vs care as usual [21].

NMB = (ΔE × WTP) − ΔC

The NMB is positive if the ICER is below the WTP threshold per QALY.

A 10-year time horizon was selected to adequately capture the long-term clinical and economic consequences of the intervention. To evaluate the robustness of the results, both deterministic and probabilistic sensitivity analyses were conducted.

The model was constructed and analyzed using Microsoft Excel 365 (version 16.77.1). The analysis was conducted in accordance with the Modeling Good Research Practices published by the ISPOR (International Society for Pharmacoeconomics and Outcomes Research) Task Force [22].

### Simulated Cohort

Eligible participants met the following inclusion criteria: adults (female, male, or nonbinary) aged 18 to 75 years, with a BMI of 30 to 45 kg/m² and a physician-confirmed diagnosis of obesity. Additional requirements included ownership of a smartphone compatible with the Oviva Direkt app, absence of severe medical conditions that would contraindicate lifestyle interventions, and no recent or planned use of antiobesity pharmacotherapy. Among enrolled participants, the median age was 46 (IQR 38-54) years, 42% (n/N) were female, and the mean body weight was 119 kg, corresponding to a mean BMI of 37.8 kg/m^2^ [14]. This means that individuals enter the model at age 46 years with the specified weight.

### Weight Development

**Figure 2:**
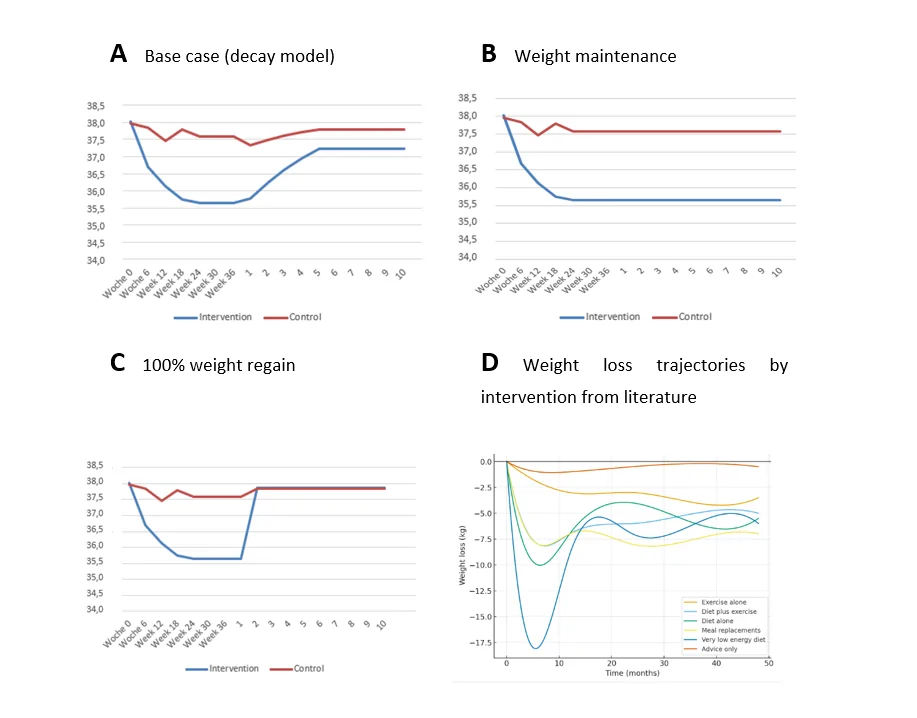
Long-term weight trajectories used in the economic model. (A) Base-case decay scenario assuming progressive weight regain beginning after year 1 and reaching 80% of the initial weight loss over 5 years (W(t) = W_baseline − (W_baseline − W_min) × e^(−k × t), where k denotes the regain rate). (B) Weight-maintenance scenario assuming that the body weight achieved at the end of the intervention is maintained over the model horizon. (C) Complete weight-regain scenario assuming progressive regain beginning after year 1 and reaching 100% of the initial weight loss. (D) Published long-term weight trajectories following lifestyle interventions, recreated from Franz et al. Pharmacological intervention groups (orlistat and sibutramine) were omitted to focus on lifestyle interventions. The published trajectories illustrate the gradual attenuation of weight loss following behavioral interventions and provide contextual support for the long-term weight trajectories evaluated in the model. The x-axis indicates time, and the y-axis denotes average BMI.

To reflect uncertainty in long-term weight trajectories following the 6-month intervention period, we modeled 3 scenarios based on the existing literature. First, a weight maintenance scenario (optimistic) assumed that the weight achieved at 6 months would remain stable over the 10-year time horizon. Second, a weight regain scenario (pessimistic) assumed that weight regain begins after the first year following the intervention, with participants progressively regaining 100% of their initial weight loss over time. Weight regain commonly begins after 1 year, often during the second year [23]. Third, a decay model assumed progressive weight regain over a period of 5 years, reaching 80% of the initial weight loss. This scenario, which served as the base-case analysis, was based on the long-term outcomes observed in the Look AHEAD trial [24]. Although Look AHEAD differed from the present intervention in terms of population and mode of delivery, both interventions represent structured multimodal lifestyle programs including dietary counseling, behavioral support, and physical activity. Furthermore, after the initial intensive intervention period, the Look AHEAD program transitioned to a maintenance phase with substantially reduced contact frequency. In the absence of long-term follow-up data for DHAs, Look AHEAD therefore represents the best available evidence to inform long-term weight trajectories. Importantly, the gradual weight regain assumed in the base case is also supported by the meta-analysis by Franz et al [23], which demonstrated that structured lifestyle interventions combining dietary and behavioral components continue to show a persistent weight difference compared with usual care for up to 36 months, despite gradual attenuation of the treatment effect [23,25]. This is illustrated in Figure 2D [23], which depicts the meta-analysis curves and demonstrates comparability with the “diet plus exercise” and “advice only” trajectories. These scenarios were incorporated to represent alternative plausible long-term weight trajectories and to evaluate the impact of different postintervention weight trajectories on long-term clinical and economic outcomes (Figure 2; Table S1 in Multimedia Appendix 2).

To link weight trajectories to clinical outcomes, we categorized individuals into 5 BMI strata: 18.5-24.9 kg/m^2^, 25.0-29.9 kg/m^2^, 30.0-34.9 kg/m^2^, 35.0-39.9 kg/m^2^, and 40.0–44.9 kg/m^2^. For each cohort (intervention and control), the distribution of individuals across these BMI categories was determined at each modeled time point. These distributions were then used to assign BMI-dependent event rates for downstream clinical outcomes within the model.

### Clinical Data Inputs

The baseline distribution of glycemic states (normoglycemia, prediabetes, and T2D) by BMI category was derived from the COM [18]. Incidence rates for key comorbidities, including T2D, ACS (MI and unstable angina), stroke, and OSA, were informed by German epidemiological data and linked to BMI-specific Cox proportional HRs for each event (Table S1 in Multimedia Appendix 3 [18,26-31]).

In line with the COM structure, obesity-associated cancers, specifically colorectal, postmenopausal breast, and endometrial cancer, were incorporated into the model. Cancer incidence rates for specific diagnoses were obtained from the COM, and HRs linking obesity to cancer risk were sourced from a recent meta-analysis by Fontvielle et al [32].

All-cause mortality was modeled using age- and sex-specific life tables for the German population. Excess mortality associated with ACS, stroke, and selected cancers was applied through adjustments to background mortality rates, based on the COM framework.

Details of all baseline distributions, transition probabilities, HRs, mortality parameters, and data sources, together with their assumed probability distributions for sensitivity analyses, are reported in Table S1 in Multimedia Appendix 3.

### Cost Assessment

The cost analysis was based on assigning health care costs to specific health states, with each state’s cost derived from the associated resource use. This included the type and frequency of medical services and goods used by patients, valued in monetary terms, to estimate total direct costs within the German health care context. Both direct and indirect costs related to obesity were incorporated into the model. Direct costs included 2 DHA prescriptions for the Oviva Direkt app (€220.90 each), treatment costs of obesity-related comorbidities, and obesity-associated cancers. These estimates were informed by published literature providing cost data specific to the German health care system. All direct costs were adjusted for inflation and standardized to 2025 euros.

Indirect costs, such as productivity losses due to sick leave and early retirement, were also included. These were stratified by BMI category and age group and applied to the working population. Productivity losses were estimated using BMI-specific odds ratios (ORs) for absenteeism and early retirement derived from a German population-based cohort analysis [7]. Parameter estimates and corresponding uncertainty ranges are reported in Table S1 in Multimedia Appendix 4 [7,33-37].

To reflect time preferences over the 10-year model horizon, future costs and outcomes were discounted at an annual rate of 3%, as recommended by the German Institute for Quality and Efficiency in Health Care (IQWiG). A detailed overview of all cost parameters is provided in Multimedia Appendix 4.

### Utility Data Inputs

Utilities quantify the preference for specific health states on a scale from 0 (death) to 1 (perfect health), reflecting their perceived value. Utility values used in the model were derived from international literature and adjusted as necessary. QALYs were calculated by multiplying the time spent in each health state by its corresponding utility, combining both length and quality of life into a single measure [38]. The model incorporates age-dependent utility values, with disutilities applied for each BMI category and associated comorbidities. For MI and stroke, event-specific disutilities are modeled: a larger utility decrement is applied in the year of the acute event, followed by a smaller, persistent disutility in subsequent post-MI and poststroke years. Comprehensive details can be found in Multimedia Appendix 5 [36,39-41].

### Sensitivity Analysis

We conducted a deterministic one-way sensitivity analysis to assess how variations in individual input parameters affect model outputs, specifically the ICER, thereby evaluating result robustness. In total, 163 model input parameters were varied across their predefined ranges. Input ranges were based on 95% CIs when available; otherwise, ranges were defined by adjusting baseline values by a fixed percentage. Additionally, a probabilistic sensitivity analysis was performed using a second-order Monte Carlo simulation, which accounts for uncertainty across all parameters simultaneously. For this analysis, input variables were assigned probability distributions: gamma for costs and beta for probabilities and utilities. A total of 1000 simulations were run, each sampling randomly from these distributions.

## Results

### Cost-Effectiveness and Clinical Outcomes

Under the base-case assumption using the decay model for weight regain, the average total cost per patient in the DHA group was €73,410.81 over a 10-year time horizon, comprising €14,723.84 in direct costs and €58,686.97 in indirect costs. In comparison, patients in the care-as-usual group incurred €76,922.67, including €15,239.74 in direct and €61,682.93 in indirect costs. The use of a DHA resulted in overall cost savings of €3511.85 per patient. Notably, direct medical costs alone were reduced by €515.90 in the intervention group.

In terms of health-related quality of life, the DHA was associated with 5.7172 QALYs per patient, compared with 5.6489 QALYs in the control group, equating to a gain of approximately 3.6 weeks in perfect health. These findings indicate that the DHA offers both economic benefits and improvements in quality of life compared to care as usual, representing a dominant strategy in the German context (Table 1).

**Table 1:** Cost-effectiveness results of the Oviva Direkt DHA^a^ under the base-case (decay model) scenario.

Model outcomes	DHA (€)	Care as usual (€)
Oviva Direkt	441.80	N/A^b^
T2D^c^ costs	9723.86	10,419.38
ACS^d^	151.01	171.35
Post ACS	840.13	924.31
Stroke	45.82	45.83
Post stroke	352. 28	352.34
OSA^e^	3137.21	3294.55
Cancer costs	31.73	31.99
Total direct costs	14,723.84	15,239.74
Sick leave costs (without cancer)	48,722.09	51,066.00
Early retirement costs	9793.02	10,444.11
Sick leave days cancer	171.86	172.82
Total indirect costs	58,686.97	61,682.93
Total costs^f^	73,410.81	76,922.67
QALYs^g^	5.7172	5.6489
LYs^h^	8.3439	8.3415

^a^DHA: digital health app.

^b^N/A: not applicable.

^c^T2D: type 2 diabetes

^d^ACS: acute coronary syndrome.

^e^OSA: obstructive sleep apnea.

^f^Cost difference: –€3511.85.

^g^QALY: quality-adjusted life years; QALY difference: 0.0683; incremental cost-utility ratio/QALY: DOMINANT (–€51,425.51); net monetary benefit: €7051.55.

^h^LY: life years; LY difference: 0.0024; incremental cost-effectiveness ratio/QALY: DOMINANT (–€2,250,976.12).

At the end of the 10-year period, the prevalence of T2D was 37.9% in the intervention group vs 39.5% in the control group, corresponding to a relative risk of 0.96 (4% relative risk reduction), an absolute risk reduction of 1.6%, and a number needed to treat of 63. This translated into an average reduction of approximately 8 months spent with T2D per patient. The prevalence of ACS was likewise lower among app users, corresponding to a relative risk of 0.79 (21% relative risk reduction) and an absolute risk reduction of 1.0%, equivalent to a number needed to treat of 100. No notable difference in stroke prevalence was observed, likely due to the relatively short time horizon and the age distribution of the simulated cohort.

Alternative weight-development scenarios confirmed the robustness of the results. Under the weight-maintenance scenario, total costs in the intervention group decreased to €66,170.59, compared with €75,845.77 in the control group, resulting in a cost saving of €9675.18 and a QALY gain of 0.2034 (equivalent to 10.6 weeks in perfect health; Table S1 in Multimedia Appendix 6). The 100% weight-regain scenario also yielded a dominant result, albeit with a reduced cost advantage of €823.28 and a smaller QALY gain equivalent to 2 days in perfect health (Table S2 in Multimedia Appendix 6).

### Deterministic Sensitivity Analysis

**Figure 3:**
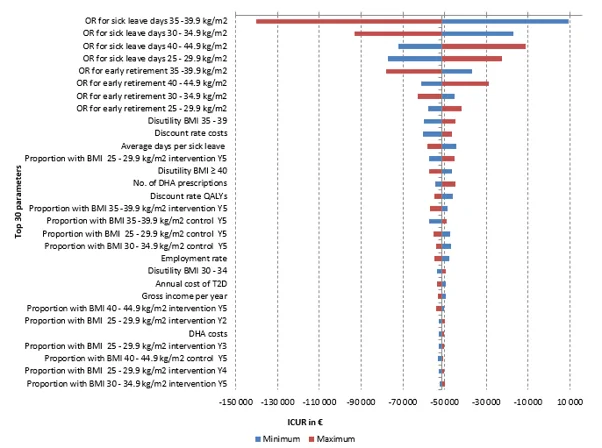
Deterministic one-way sensitivity analysis of the incremental cost-utility ratio (ICUR) from the societal perspective, visualized as tornado plots. Deterministic sensitivity analysis was used to identify the critical variables affecting risk analysis. Results are displayed as tornado diagrams for the 30 variations with the greatest impact, where each bar represents a one-way sensitivity analysis, and the width of the bars represents impact on model results. The ICUR is plotted on the x-axis. DHA: digital health app; OR: odds ratio; T2D: type 2 diabetes.

A one-way sensitivity analysis was conducted to assess the impact of variations in individual model parameters on the incremental cost-utility ratio (ICUR). Results from the societal perspective are presented in Figure 3. The analysis revealed that the OR for sick leave had the greatest influence on the ICUR. This is primarily due to the fact that indirect costs, driven largely by productivity losses, substantially outweigh direct medical costs, making parameters related to absenteeism particularly impactful.

From the payer’s perspective, the five most influential parameters were (1) number of DHA prescriptions, (2) baseline age of the cohort, (3) annual cost of managing T2D, (4) the discount rate applied to costs, and (5) the proportion of patients with BMI 40 to 44.9 kg/m^2^ in the intervention group in year 5 (Figure S1 in Multimedia Appendix 7). This indicates that model outcomes are primarily driven by demographic structure and reimbursement-related parameters rather than clinical effect sizes. Importantly, the number of DHA prescriptions, which depends on policy and uptake in routine practice, emerged as a key driver of cost-effectiveness.

These findings underscore the relevance of population characteristics and long-term disease burden in determining cost-effectiveness outcomes across different perspectives. In particular, the prominence of the annual cost of T2D among the most influential parameters from the payer perspective highlights the importance of obesity-related comorbidities in driving long-term health care costs.

### Probabilistic Sensitivity Analysis

**Figure 4:**
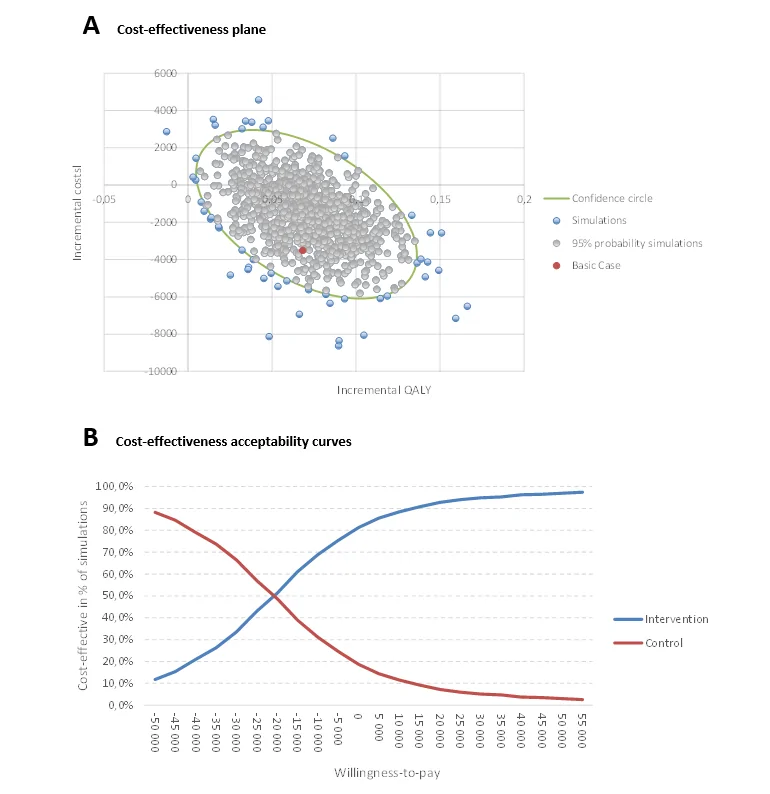
Probabilistic sensitivity analysis from the societal perspective: (A) Cost-effectiveness plane showing the results of the Monte Carlo probabilistic sensitivity analysis for 1000 patients. Incremental cost is plotted on the y-axis, and incremental effectiveness is plotted on the x-axis. (B) Cost-effectiveness acceptability curves display the percentage of iterations that favor the use of a DHA in comparison to care as usual over a range of WTP. The x-axis displays reported values in € per quality-adjusted life-year (QALY).

The ICER scatterplot, representing incremental costs on the y-axis and incremental QALYs on the x-axis, portrays the cost-utility analysis outcomes. It reveals that the use of a DHA compared to care as usual is, in the majority of cases, less costly (81.2%) yet more effective when expressed in QALYs; the majority of points are clustered in the lower right quadrant (Figure 4A). The average incremental cost amounts to –€1565.18, while the incremental QALYs reach 0.070. This yields a probabilistic ICUR of –€22,207.24, indicating dominance (Figure 4A).

The acceptability curve indicates that when considering a WTP threshold of €51,833 per QALY gained, the DHA vs care as usual is deemed cost-effective in 97% of instances. Even with a WTP threshold of €25,000, 94% of all simulations are in the cost-effective range (Figure 4B).

From the payer’s perspective, all simulations fall within the lower right quadrant of the cost-effectiveness plane, indicating that the intervention is both more effective and less costly, that is, a dominant strategy. The average incremental cost across simulations was –€532.22, resulting in an ICUR of –€7551.27 per QALY gained (Figure S2 in Multimedia Appendix 7).

## Discussion

### Principal Findings

The increasing prevalence of obesity and its comorbidities represents a major challenge for health systems worldwide, both clinically and economically. In Germany, approximately 10% of all health care expenditures are attributable to obesity-related diseases, with total direct and indirect costs estimated at around 63 billion euros annually [6,42]. These costs are largely driven by chronic comorbidities such as T2D, cardiovascular disease, and certain forms of cancer. Against this background, scalable and cost-effective prevention strategies are urgently needed.

This study evaluated the long-term cost-effectiveness of Oviva Direkt in the German context using a Markov cohort model over a 10-year time horizon.

From the payer’s perspective, the intervention was already cost-saving. When viewed from the broader societal perspective, the impact was more pronounced, reflecting the importance of including productivity losses and early retirement in economic evaluations. This highlights that digital interventions not only generate savings for health insurers but also deliver substantial value to society as a whole. While a formal budget impact analysis was beyond the scope of this study, the estimated societal cost savings of €3511.85 per treated patient provide an indication of the potential economic impact of wider implementation. Based on approximately 300,000 patients receiving Oviva Direkt for obesity, as reported in the 2025 DiGA Report [43], cumulative societal savings could exceed €1.05 billion over a 10-year period, assuming comparable treatment effects and long-term outcomes. These estimates are intended to be illustrative and should be interpreted with caution, as actual savings will depend on patient characteristics, adherence, and the durability of treatment effects in routine clinical practice.

Pricing remains an important issue. While statutory health insurers and DHA providers may have conflicting interests with respect to reimbursement levels, robust evidence of effectiveness and cost savings can strengthen the case for fair pricing models. Interestingly, varying the assumed price of the DHA had only a limited influence on model outcomes from a societal perspective in the sensitivity analysis, suggesting that the long-term economic value of such interventions depends more on their ability to reduce the burden of chronic disease than on short-term pricing assumptions.

The model outcomes are inherently linked to assumptions about weight trajectories. In the base-case decay scenario, the initial BMI benefit observed after the 6-month intervention gradually diminished over time due to progressive weight regain, while remaining below that of the control group throughout the model horizon. Consequently, the projected QALY and life-year gains were modest and primarily accrued during the period of lower BMI, when individuals experienced a reduced risk of obesity-related comorbidities and improved health-related quality of life. Even in the complete weight-regain scenario, temporary differences in BMI delayed the onset of obesity-related diseases, particularly T2D, thereby reducing cumulative years lived with disease and associated health care costs over the 10-year time horizon. In the absence of long-term follow-up data for DHAs, the decay trajectory was informed by the Look AHEAD trial and supported by evidence from the meta-analysis by Franz et al [23], indicating that structured lifestyle interventions are generally characterized by gradual, rather than immediate, weight regain [23,24]. Even when weight loss is not consistently statistically significant over the long term, reductions achieved in the early years are sufficient to lower health care resource use and direct medical costs. More importantly, they also influence indirect costs, such as productivity losses, which represent a major share of the overall economic burden. Sensitivity analyses confirmed this pattern: from the societal perspective, the odds ratio for sick leave exerted the greatest influence on the ICUR, underlining the substantial role of indirect costs, such as productivity losses. From the payer’s perspective, the 5 most influential drivers were the number of DHA prescriptions, the baseline cohort age, the annual cost of managing T2D, the applied discount rate, and the proportion of patients with BMI 40 to 44.9 kg/m^2^ at year 5 (Figures 3 and Table S1 in Multimedia Appendix 1). Together, these findings suggest that cost-effectiveness is primarily shaped by demographic and epidemiological factors and the rising costs of chronic disease management, particularly diabetes, in the future. The strong impact of diabetes-related costs reflects not only the increasing prevalence of the disease but also the trend that newer therapies are consistently more expensive than those available in the past.

While the focus of this study was obesity, other digital health interventions have also demonstrated economic and clinical benefits in different disease contexts. For instance, Freitag et al [44] evaluated mHealth apps for the treatment of depression in Germany and found that under the current cost structure they were not cost-effective compared to standard care but improved patient outcomes, using a similar Markov cohort model. Likewise, Walter et al [45] reported that the Floodlight MS app, designed to monitor disease progression in patients with multiple sclerosis, provided clinical and economic benefits from the perspective of the Austrian health care system. These findings support the broader health economic potential of digital apps across a range of chronic diseases.

Taken together, these studies, including the present one, underline the value of digital tools in addressing the burden of chronic disease by promoting self-management, prevention, and early intervention. Particularly in the context of obesity, where treatment options are often resource-intensive and long-term engagement is crucial, apps like Oviva Direkt can offer a scalable and cost-saving complement to traditional care. Given the projected increase in obesity prevalence and its associated costs, investing in such digital solutions may not only benefit individual patients but also reduce the long-term burden on public health systems.

This study has several strengths. First, it is among the first cost-effectiveness analyses to evaluate a digital lifestyle intervention for obesity treatment, specifically tailored to the German health care system. The use of a Markov cohort model with BMI stratification and the integration of both direct and indirect cost components enables a comprehensive estimation of the economic and clinical impact of the intervention. The model structure and clinical parameters were informed by established models (such as COM) and updated using the most recent epidemiological and meta-analytical data [32]. Furthermore, the incorporation of alternative weight trajectories (maintenance and regain) supports the robustness of the results.

Importantly, this analysis accounts for long-term health outcomes and costs over a 10-year horizon and applies a 3% annual discount rate, in line with recommendations by IQWiG. By restricting the horizon to 10 years, however, costs and benefits that would occur later in life are truncated, potentially underestimating the long-term value of the intervention. To illustrate the impact of extending the time horizon, we conducted a 20-year scenario, in which the intervention remained dominant, with incremental direct costs of –€2026.67 from the payer perspective and –€6436.94 from the societal perspective (direct and indirect costs). The inclusion of indirect costs, such as productivity losses and early retirement, reflects the broader societal perspective and highlights the economic burden of obesity beyond health care expenditures alone.

Nevertheless, some limitations should be acknowledged. As with any modeling study, the validity of the results depends on the accuracy of the input data and assumptions. Although the clinical inputs were derived from German population data where possible, some assumptions, such as disease progression, utility values, or cancer risks, had to be informed by international literature or model defaults because of limited local data availability. In addition, while the model assumes differential effects on BMI over time (decay, regain, and maintenance), real-world adherence to and long-term engagement with digital interventions may vary and are difficult to predict accurately. A key uncertainty relates to the extrapolation of long-term weight trajectories. Because long-term follow-up data for DHAs are currently unavailable, the base-case analysis was informed by the Look AHEAD trial, representing the longest available follow-up of a structured multimodal lifestyle intervention. Although differences in population and delivery format exist, both interventions comprise the core components of behavioral obesity management, including dietary counseling, behavioral support, and physical activity. Furthermore, the gradual weight-regain trajectory observed in Look AHEAD is consistent with the broader evidence from the meta-analysis by Franz et al [23], demonstrating that structured lifestyle interventions are generally characterized by progressive, rather than complete, weight regain, with persistent weight differences compared with usual care for up to 36 months [23]. Therefore, Look AHEAD was considered the best available evidence to inform long-term weight trajectories in the absence of long-term follow-up data for digital interventions. Nevertheless, uncertainty regarding long-term weight development remains. To address this uncertainty, additional optimistic (weight maintenance) and pessimistic (100% weight regain) scenario analyses were performed to evaluate the impact of alternative long-term weight trajectories on the model results.

The comparator in the present model is “care as usual,” as implemented in the underlying randomized controlled trial. As obesity management in Germany is heterogeneous and continues to evolve, including the increasing use of glucagon-like peptide-1 (GLP-1) receptor agonists, the present results should be interpreted as the incremental benefit of adding a DHA to routine care rather than as a comparison with all currently available obesity management strategies. Importantly, current obesity management guidelines recommend structured multimodal lifestyle intervention, including dietary modification, physical activity, and behavioral support, as the foundation of obesity treatment, with pharmacological therapies generally considered part of a stepwise treatment escalation for eligible patients [19]. The present analysis therefore reflects this stage of the treatment pathway rather than a comparison with pharmacological obesity therapies. Future studies should evaluate DHAs in comparison with alternative treatment approaches, including GLP-1 receptor agonists and other pharmacological obesity therapies.

This model did not include the full spectrum of obesity-related comorbidities. Conditions such as depression, musculoskeletal disorders, and orthopedic indications, frequently reported in cost-effectiveness studies of obesity, were excluded due to limited availability of data. In addition, 3 major obesity-associated cancers were included, while other neoplasms linked to obesity were not considered. Their omission likely results in an underestimation of the quality-of-life and economic benefits, making our findings a conservative estimate of the true value.

Furthermore, the model does not include potential long-term complications beyond the 10-year horizon, such as microvascular complications of T2D, nor does it account for intangible benefits of behavioral change (eg, improved mental health or social participation), potentially underestimating the full value of the intervention. Bariatric surgery is associated with substantial costs for payers while also affecting long-term QALYs [46]. The omission of such factors suggests that our analysis may underestimate the total future costs of obesity.

For this analysis, we conservatively assumed 2 DHA prescriptions per patient, reflecting the initial prescription and 1 renewal as foreseen in the current German framework. While real-world uptake patterns show considerable variability, often being lower because of limited adherence, longer or repeated use beyond 2 prescriptions could further enhance effectiveness but would also increase costs. Our assumption therefore provides a balanced and likely conservative estimate of both costs and benefits. In addition, the model did not explicitly account for real-world adherence or dropout. Clinical effectiveness was based on the observed weight change in the underlying randomized controlled trial and therefore reflects adherence patterns within the trial setting rather than routine clinical practice. Lower engagement under real-world conditions could reduce both the clinical effectiveness and the economic value of the intervention. Although the deterministic sensitivity analysis explored variation in the number of DHA prescriptions as a pragmatic proxy for differences in treatment persistence, the weight-regain scenarios capture uncertainty regarding the persistence of treatment effects over time and should not be interpreted as a substitute for explicit adherence-based modeling. Future studies incorporating real-world evidence on DHA uptake, treatment persistence, and completion rates would further strengthen the evidence base for long-term economic evaluations.

Finally, the digital health space evolves rapidly, and changes in pricing, reimbursement structures, or technological features of interventions like Oviva Direkt may influence future cost-effectiveness outcomes.

### Conclusion

In conclusion, this study provides evidence that the use of the Oviva Direkt app is not only clinically beneficial but also economically advantageous in the German health care setting. By achieving better outcomes at lower cost, it represents a dominant strategy for obesity management. These results align with a growing body of literature suggesting that DHAs can play a pivotal role in chronic disease prevention and care, offering sustainable and patient-centered solutions to some of the most pressing public health challenges of our time. Taken together, these findings support the integration of evidence-based digital interventions into routine obesity care and highlight the need for further research on long-term effectiveness, adherence, and implementation across diverse health care systems.

## Data Availability

All data analyzed during this study are included in this published article and its supplementary information files. Clinical effectiveness data were derived from the published randomized controlled trial evaluating the Oviva Direkt digital health application for weight management (DRKS00033045; Lautenbach et al [14]), while all remaining model inputs were obtained from published sources.

## Appendix

Multimedia Appendix 1 Clinical trial data.

Multimedia Appendix 2 Weight development (base case and decay model).

Multimedia Appendix 3 Transition probabilities and clinical input data.

Multimedia Appendix 4 S4 Cost assessment.

Multimedia Appendix 5 Health state utilities.

Multimedia Appendix 6 Cost-effectiveness results: optimistic and pessimistic scenarios.

Multimedia Appendix 7 Sensitivity analyses from the payer perspective.

## Abbreviations

ACS: acute coronary syndrome
COM: Core Obesity Model
DHA: digital health app
DiGA: Digital Health Applications
GLP-1: glucagon-like peptide-1
HR: hazard ratio
ICER: incremental cost-effectiveness ratio
ICUR: incremental cost-utility ratio
IQWiG: Institute for Quality and Efficiency in Health Care
mHealth: mobile health
MI: myocardial infarction
NMB: net monetary benefit
OR: odds ratio
OSA: obstructive sleep apnea
QALY: quality-adjusted life-year
T2D: type 2 diabetes
WTP: willingness-to-pay
