# Supplementary material for: Long-Term Cost and Health Impact of a Digital Obesity Intervention in Germany: Economic Modeling Study
Source: JMIR Form Res. 2026 Aug 24;10:e88576. doi: 10.2196/88576 (PMC13550978; doi:10.2196/88576)
Supplement: Supplementary file 1 [file formative_v10i1e88576_app1.docx]

**Multimedia Appendix 1**

*Clinical Trial Data*

The economic model was informed by data from the published randomized controlled trial by Lautenbach et al (2026) [1] evaluating the effectiveness of the Oviva Direkt digital health application in adults with obesity in Germany (DRKS00033045). Detailed information on the study design, recruitment procedures, eligibility criteria, baseline characteristics, participant flow, and clinical outcomes is provided in the original publication.

For the present economic evaluation, the trial was used to parameterize the short-term treatment effect on body weight and BMI. Specifically, the observed body weight and BMI values at each study visit were used to estimate treatment-specific BMI distributions and weight trajectories during the intervention period. Because these numerical values are not reported in the original publication, they are summarized below to facilitate reproducibility of the economic model.

**Table S1.** Body weight and BMI over time.

| Time point | Intervention (n) | Mean weight (kg) Intervention | Mean BMI (kg/m²) Intervention | Controll (n) | Mean weight (kg) Control | Mean BMI (kg/m²) Control |
| --- | --- | --- | --- | --- | --- | --- |
| Week 0 | 85 | 118.98 | 37,72 | 79 | 118.76 | 37,93 |
| Week 6 | 66 | 115.42 | 36,61 | 68 | 118.60 | 37,69 |
| Week 12 | 61 | 113.95 | 36,47 | 59 | 117.31 | 37,33 |
| Week 18 | 57 | 112.07 | 36,09 | 63 | 118.83 | 37,64 |
| Week 24 | 59 | 111.02 | 35,91 | 62 | 118.50 | 36,96 |

**References**

1. Lautenbach A, Blüher M, Mijuskovic A, Jones L, Schirmann F. Efficacy of a digital health application for weight management in people with obesity: 6-months results from a randomized controlled trial. Int J Obes (Lond). 2026 Feb;50(2):466-473. doi: 10.1038/s41366-025-01967-3. Epub 2025 Dec 17. PMID: 41408456; PMCID: PMC12913030.
