# Supplementary material for: Long-Term Cost and Health Impact of a Digital Obesity Intervention in Germany: Economic Modeling Study
Source: JMIR Form Res. 2026 Aug 24;10:e88576. doi: 10.2196/88576 (PMC13550978; doi:10.2196/88576)
Supplement: Supplementary file 2 [file formative_v10i1e88576_app2.docx]

**Multimedia Appendix 2**

**Table S1.** Weight development (base case and decay model).

| **Weight development intervention** | | | | |
| --- | --- | --- | --- | --- |
| Proportion with BMI 18.5 - 24.9 kg/m2 Y1  Proportion with BMI 25 - 29.9 kg/m2 Y1  Proportion with BMI 30 - 34.9 kg/m2 Y1  Proportion with BMI 35 -39.9 kg/m2 Y1  Proportion with BMI 40 - 44.9 kg/m2 Y1  Proportion with BMI 18.5 - 24.9 kg/m2 Y2  Proportion with BMI 25 - 29.9 kg/m2 Y2  Proportion with BMI 30 - 34.9 kg/m2 Y2  Proportion with BMI 35 -39.9 kg/m2 Y2  Proportion with BMI 40 - 44.9 kg/m2 Y2  Proportion with BMI 18.5 - 24.9 kg/m2 Y3  Proportion with BMI 25 - 29.9 kg/m2 Y3  Proportion with BMI 30 - 34.9 kg/m2 Y3  Proportion with BMI 35 -39.9 kg/m2 Y3  Proportion with BMI 40 - 44.9 kg/m2 Y3  Proportion with BMI 18.5 - 24.9 kg/m2 Y4  Proportion with BMI 25 - 29.9 kg/m2 Y4  Proportion with BMI 30 - 34.9 kg/m2 I4  Proportion with BMI 35 -39.9 kg/m2 Y4  Proportion with BMI 40 - 44.9 kg/m2 Y4  Proportion with BMI 18.5 - 24.9 kg/m2 Y5  Proportion with BMI 25 - 29.9 kg/m2  Proportion with BMI 30 - 34.9 kg/m2 Y5  Proportion with BMI 35 -39.9 kg/m2 Y5  Proportion with BMI 40 - 44.9 kg/m2 Y5 | 7,5%  21,6%  30,4%  26,7%  13,8%  6,1%  20,6%  30,1%  26,0%  17,2%  4,9%  19,7%  29,9%  25,5%  20,0%  3,9%  19,0%  29,7%  25,0%  22,4%  3,1%  18,3%  29,6%  24,6%  24,4% | ± 10%  ± 10%  ± 10%  ± 10%  ± 10%  ± 10%  ± 10%  ± 10%  ± 10%  ± 10%  ± 10%  ± 10%  ± 10%  ± 10%  ± 10%  ± 10%  ± 10%  ± 10%  ± 10%  ± 10%  ± 10%  ± 10%  ± 10%  ± 10%  ± 10% | BETA  BETA  BETA  BETA  BETA  BETA  BETA  BETA  BETA  BETA  BETA  BETA  BETA  BETA  BETA  BETA  BETA  BETA  BETA  BETA  BETA  BETA  BETA  BETA  BETA | Decay model estimation |
| **Weight development control** | | | | |
| Proportion with BMI 18.5 - 24.9 kg/m2 Y1  Proportion with BMI 25 - 29.9 kg/m2 Y1  Proportion with BMI 30 - 34.9 kg/m2 Y1  Proportion with BMI 35 -39.9 kg/m2 Y1  Proportion with BMI 40 - 44.9 kg/m2 Y1  Proportion with BMI 18.5 - 24.9 kg/m2 Y2  Proportion with BMI 25 - 29.9 kg/m2 Y2  Proportion with BMI 30 - 34.9 kg/m2 Y2  Proportion with BMI 35 -39.9 kg/m2 Y2  Proportion with BMI 40 - 44.9 kg/m2 Y2  Proportion with BMI 18.5 - 24.9 kg/m2 Y3  Proportion with BMI 25 - 29.9 kg/m2 Y3  Proportion with BMI 30 - 34.9 kg/m2 Y3  Proportion with BMI 35 -39.9 kg/m2 Y3  Proportion with BMI 40 - 44.9 kg/m2 Y3  Proportion with BMI 18.5 - 24.9 kg/m2 Y4  Proportion with BMI 25 - 29.9 kg/m2 Y4  Proportion with BMI 30 - 34.9 kg/m2 I4  Proportion with BMI 35 -39.9 kg/m2 Y4  Proportion with BMI 40 - 44.9 kg/m2 Y4  Proportion with BMI 18.5 - 24.9 kg/m2 Y5  Proportion with BMI 25 - 29.9 kg/m2  Proportion with BMI 30 - 34.9 kg/m2 Y5  Proportion with BMI 35 -39.9 kg/m2 Y5  Proportion with BMI 40 - 44.9 kg/m2 Y5 | 5,4%  10,6%  39,6%  26,2%  18,2%  4,7%  11,0%  37,6%  28,4%  18,3%  4,1%  11,3%  35,8%  30,4%  18,4%  3,6%  11,6%  34,2%  32,1%  18,5%  3,2%  11,9%  32,8%  33,6%  18,5% | ± 10%  ± 10%  ± 10%  ± 10%  ± 10%  ± 10%  ± 10%  ± 10%  ± 10%  ± 10%  ± 10%  ± 10%  ± 10%  ± 10%  ± 10%  ± 10%  ± 10%  ± 10%  ± 10%  ± 10%  ± 10%  ± 10%  ± 10%  ± 10%  ± 10% | BETA  BETA  BETA  BETA  BETA  BETA  BETA  BETA  BETA  BETA  BETA  BETA  BETA  BETA  BETA  BETA  BETA  BETA  BETA  BETA  BETA  BETA  BETA  BETA  BETA | Decay Model estimation |
