# Supplementary material for: Long-Term Cost and Health Impact of a Digital Obesity Intervention in Germany: Economic Modeling Study
Source: JMIR Form Res. 2026 Aug 24;10:e88576. doi: 10.2196/88576 (PMC13550978; doi:10.2196/88576)
Supplement: Supplementary file 3 [file formative_v10i1e88576_app3.docx]

**Multimedia Appendix 3**

*Transition probabilities and clinical input data*

The baseline distribution of glycaemic states (normoglycaemia, prediabetes, and type-2-diabetes [T2D]) by BMI category was not derived from the clinical trial or German epidemiological data, but rather sourced from UK data within the Core Obesity Model (COM) as reported by Lopes et al. (2021). Incidence rates for T2D were obtained from Tamayo et al. (2016), who analyzed health insurance claims from 6.4 million individuals in 2009 and 6.7 million in 2010, out of approximately 65 million insured persons in Germany. The authors provided age- and sex-standardized incidence rates, which were directly applied in the model.

For acute coronary syndrome (ACS; including myocardial infarction and unstable angina) and stroke, only prevalence data were available. Therefore, incidence rates were estimated using the formula:

incidence = –ln(1 – prevalence) / t,

assuming a steady-state population over time. The prevalence data were sourced from the "Study on the Health of Adults in Germany" (DEGS), conducted by the Robert Koch Institute (RKI), as published by Größwald et al. (2013) and Busch et al. (2013).

The prevalence of obstructive sleep apnoea (OSA) was based on findings from the population-based Study of Health in Pomerania (SHIP), which investigated clinical conditions and risk factors in northeastern Germany. Fietze et al. (2019) reported an OSA prevalence of 21% overall (30% in men, 13% in women), using an apnea–hypopnea index (AHI) threshold of ≥15 events per hour. Incidence rates were again derived using the aforementioned transformation formula.

The model links BMI-specific risks for comorbidities using Cox proportional hazard ratios (HRs) obtained from the COM (Lopes et al., 2021). For OSA specifically, an elevated risk was assumed for individuals with a BMI ≥30 kg/m², in line with published estimates (Lopes et al., 2008).

Consistent with the COM structure, the model includes obesity-related cancers—colorectal, postmenopausal breast, and endometrial cancer. Cancer incidence rates were taken from the COM (Olivieri et al., 2024), while HRs linking obesity to cancer risk were sourced from a recent meta-analysis by Fontvielle et al. (2023).

All-cause mortality was modeled using age- and sex-specific German life tables. Disease-specific excess mortality for ACS and stroke was incorporated by adjusting the background mortality, based on estimates from the COM. Cancer-related mortality during the first year after diagnosis was also based on COM inputs. In the following years, a reduced mortality rate was used. (Olivieri et al., 2024).

**Table S1.** Input data.

| **Parameter** | **Base Case** | **Range** | **Distribution** | **Source** |
| --- | --- | --- | --- | --- |
| Age  Proportion female | 46  42.1% | 40-56  ± 10% | NORMAL  BETA | Oviva trial |
| **Base Case distribution** | | | | |
| Pre-T2D with 18.5 - 24.9 kg/m2  T2D with 18.5 - 24.9 kg/m3  Pre-T2D with 25 - 29.9 kg/m2  T2D with 25 - 29.9 kg/m2  Pre-T2D with 30 - 34.9 kg/m2  T2D with 30 - 34.9 kg/m2  Pre-T2D with 35 -39.9 kg/m2  T2D with 35 -39.9 kg/m2  Pre-T2D with 40 - 44.9 kg/m2  T2D with 40 - 44.9 kg/m2 | 2,0%  2,7%  4,1%  5,7%  5,9%  8,6%  6,9%  10,6%  6,7%  14,5% | ± 10%  ± 10%  ± 10%  ± 10%  ± 10%  ± 10%  ± 10%  ± 10%  ± 10%  ± 10%  ± 10% | BETA  BETA  BETA  BETA  BETA  BETA  BETA  BETA  BETA  BETA | Lopes et al. 2021 |
| **Incidence of comorbidities** | | | | |
| Incidence ACS normal weight age 40-49  Incidence ACS normal weight age 50-59  Incidence ACS normal weight age 60-69  Incidence ACS normal weight age >70  Incidence stroke normal weight age 40-49  Incidence stoke normal weight age 50-59  Incidence stroke normal weight age 60-69  Incidence stroke normal weight age >70  Incidence T2D normal weight age 40-49  Incidence T2D normal weight age 50-59  Incidence T2D normal weight age 60-69  Incidence T2D normal weight age >70  Incidence OSA normal weight age 40-49  Incidence OSA normal weight age 50-59  Incidence OSA normal weight age 60-69  Incidence OSA normal weight age >70  Probability of ACS is MI age 40-49  Probability of ACS is MI age 50-59  Probability of ACS is MI age 60-69  Probability of ACS is MI age ≥ 70 | 0,23%  0,45%  1,64%  2,52%  0,11%  0,13%  0,43%  0,74%  0,32%  0,77%  1,59%  2,39%  0,38%  0,53%  0,89%  2,67%  44,12%  32,79%  40,39%  33,89% | ± 10%  ± 10%  ± 10%  ± 10%  ± 10%  ± 10%  ± 10%  ± 10%  ± 10%  ± 10%  ± 10%  ± 10%  ± 10%  ± 10%  ± 10%  ± 10%  ± 10%  ± 10%  ± 10%  ± 10% | BETA  BETA  BETA  BETA  BETA  BETA  BETA  BETA  BETA  BETA  BETA  BETA  BETA  BETA  BETA  BETA  BETA  BETA  BETA  BETA | Größwald et al. 2013  Busch et al. 2013  Tamayo et al. 2016  Fietze et al. 2019  Größwald et al. 2013 |
| **Cox proportional HR used in COM** | | | | |
| HR incidence 25 - 29.9 kg/m2 ACS  HR incidence 25 - 29.9 kg/m2 stroke  HR incidence 25 - 29.9 kg/m2 T2D  HR incidence 25 - 29.9 kg/m2 mortality  HR incidence 30 - 34.9 kg/m2 ACS  HR incidence 30 - 34.9 kg/m2 stroke  HR incidence 30 - 34.9 kg/m2 T2D  HR incidence 30 - 34.9 kg/m2 mortality  HR incidence 35 -39.9 kg/m2 ACS  HR incidence 35 -39.9 kg/m2 stroke  HR incidence 35 -39.9 kg/m2 T2D  HR incidence 35 -39.9 kg/m2 mortality  HR incidence 40 - 44.9 kg/m2 ACS  HR incidence 40 - 44.9 kg/m2 stroke  HR incidence 40 - 44.9 kg/m2 T2D  HR incidence 40 - 44.9 kg/m2 mortality  HR incidence 30 - 34.9 kg/m2 OSA  HR incidence 35 - 39.9 kg/m2 OSA  HR incidence 40 - 44.9 kg/m2 OSA | 1,03  0,92  2,20  0,77  1,11  0,94  4,73  0,81  1,14  0,98  7,81  0,95  1,18  1,02  10,80  1,21  1,46  5,06  6,77 | ± 10%  ± 10%  ± 10%  ± 10%  ± 10%  ± 10%  ± 10%  ± 10%  ± 10%  ± 10%  ± 10%  ± 10%  ± 10%  ± 10%  ± 10%  ± 10%  ± 10%  ± 10%  ± 10% | GAMMA  GAMMA  GAMMA  GAMMA  GAMMA  GAMMA  GAMMA  GAMMA  GAMMA  GAMMA  GAMMA  GAMMA  GAMMA  GAMMA  GAMMA  GAMMA  GAMMA  GAMMA  GAMMA | Lopes et al. 2021  Lopes et al. 2008 |
| **Mortality due to comorbidities** | | | | |
| Mortality rate in the year of event ACS  Mortality rate in the year of event stroke  RR of mortality in years post onset ACS  RR of mortality in years post onset stroke | 31,2%  20,3%  1,30  2,00 | ± 10%  ± 10%  ± 10%  ± 10% | BETA  BETA  GAMMA  GAMMA | Lopes et al. 2021 |
| **Cancer** | | | | |
| Prevalence CC  Prevalence EC  Prevalence BC  HR for cancer obes without pre-T2D, T2D and comorbidities  HR for cancer obes with T2D  HR for cancer obes with CVD (ACS and stoke) | 0,08%  0,05%  0,20%  1,11  1,11  1,17 | ± 10%  ± 10%  ± 10%  ± 10%  ± 10%  ± 10% | BETA  BETA  BETA  GAMMA  GAMMA  GAMMA | Olivieri et al., 2024  Fontvieille et al. 2023 |
| **Cancer Mortality** | | | | |
| CC mortality in the year of onset  EC mortality in the year of onset  BC mortality in the year of onset  Cancer mortality (% in years 2+ from onset) | 30,10%  10,50%  4,10%  4,03% | ± 10%  ± 10%  ± 10%  ± 10% | BETA  BETA  BETA  BETA | Olivieri et al., 2024 |

**References**

1. Busch MA, Schienkiewitz A, Nowossadeck E, Gößwald A. Prevalence of stroke in adults aged 40–79 years in Germany Results of the German Health Interview and Examination Survey for Adults (DEGS1). Bundesgesundheitsblatt  Gesundheitsforschung - Gesundheitsschutz 5/6 · 2013
2. Fietze I, Laharnar N, Obst A, Ewert R, Felix SB, Garcia C, Gläser S, Glos M, Schmidt CO, Stubbe B, Völzke H, Zimmermann S, Penzel T. Prevalence and association analysis of obstructive sleep apnea with gender and age differences - Results of SHIP-Trend. J Sleep Res. 2019 Oct;28(5):e12770. doi: 10.1111/jsr.12770. Epub 2018 Oct 1. PMID: 30272383.
3. Gößwald A,  Schienkiewitz A,  Nowossadeck E, · Busch MA. Prävalenz von Herzinfarkt und koronarer Herzkrankheit bei Erwachsenen im Alter von 40 bis 79 Jahren in Deutschland Ergebnisse der Studie zur Gesundheit Erwachsener in Deutschland (DEGS1). Bundesgesundheitsblatt  Gesundheitsforschung - Gesundheitsschutz 5/6 · 2013
4. Lopes S, Meincke HH, Lamotte M, Olivieri AV, Lean MEJ. A novel decision model to predict the impact of weight management interventions: The Core Obesity Model. Obes Sci Pract. 2021 Mar 9;7(3):269-280. doi: 10.1002/osp4.495. PMID: 34123394; PMCID: PMC8170577.
5. Lopez PP, Stefan B, Schulman CI, Byers PM. Prevalence of sleep apnea in morbidly obese patients who presented for weight loss surgery evaluation: more evidence for routine screening for obstructive sleep apnea before weight loss surgery. Am Surg. 2008 Sep;74(9):834-8. PMID: 18807673.
6. Olivieri AV, Muratov S, Larsen S, Luckevich M, Chan K, Lamotte M, Lau DCW. Cost-effectiveness of weight-management pharmacotherapies in Canada: a societal perspective. Int J Obes (Lond). 2024 May;48(5):683-693. doi: 10.1038/s41366-024-01467-w. Epub 2024 Jan 31. PMID: 38291203; PMCID: PMC11058048.
7. Tamayo T, Brinks R, Hoyer A, Kuß O, Rathmann W. The Prevalence and Incidence of Diabetes in Germany. Deutsches Ärzteblatt International | Dtsch Arztebl Int 2016; 113: 177–82
