# Supplementary material for: Long-Term Cost and Health Impact of a Digital Obesity Intervention in Germany: Economic Modeling Study
Source: JMIR Form Res. 2026 Aug 24;10:e88576. doi: 10.2196/88576 (PMC13550978; doi:10.2196/88576)
Supplement: Supplementary file 4 [file formative_v10i1e88576_app4.docx]

**Multimedia Appendix 4**

*Cost assessment*

Both direct and indirect costs associated with obesity were incorporated into the model.

Direct costs included two reimbursed digital prescriptions of the Oviva Direkt app (€220.90 each), as well as annual treatment costs for obesity-related comorbidities and obesity-associated cancers. Cost inputs were drawn from published sources specific to the German healthcare context. Estimates for comorbidity-related expenditures were primarily based on the cost–utility analysis of bariatric surgery in Germany by Borisenko et al. (2017), with base-year costs from 2012. These were adjusted to 2025 values using the Health Consumer Price Index (HCPI) published by Destatis.

No German cost data were available for obstructive sleep apnea (OSA); thus, costs were identified through a systematic review by Alakörkkö et al. (2023) and transferred from Danish estimates to the German context using OECD purchasing power parity (PPP) adjustments.

Costs associated with obesity-related cancers (colorectal, postmenopausal breast, and endometrial) were derived from various German sources. For sex-specific cancers, incidence rates were applied only to the relevant proportion of the cohort. Postmenopausal breast cancer and endometrial cancer were therefore modelled exclusively among female participants, whereas colorectal cancer was modelled in both sexes. The proportion of female participants was based on the clinical trial population and was varied in sensitivity analyses. These differentiate between initial treatment year costs—which include surgical procedures and systemic therapies—and lower follow-up costs in subsequent years.

Indirect costs, including productivity losses due to sick leave and early retirement, were substantial and modeled by BMI category and age group, limited to the working-age population. Estimates were based on a German burden-of-disease analysis by Yates et al. (2016), which reported BMI-stratified odds ratios (ORs) for absenteeism and early retirement using data from five KORA cohort studies. These ORs were applied to the national average of 14.8 sick leave days per employee (Destatis) and adjusted by sex- and age-specific employment rates. Productivity loss was valued using the average gross annual salary in Germany (€56,412; Destatis).

For obesity-related cancers, work loss was estimated based on ICD-10-specific absenteeism data from the German Federal Ministry of Health, multiplied by the average gross wage to quantify the corresponding productivity loss.

**Table S1.** Cost data.

| **Parameter** | **Base Cas (costs in €)e** | **Range** | **Distribution** | **Source** |
| --- | --- | --- | --- | --- |
| **Direct cost inputs** | | | | |
| DHA costs  No. of DHA prescriptions  Annual cost of T2D  Annual cost of acute stroke  Annual cost of post-stroke  Cost of acute myocardial infarction  Annual cost of post-MI state  Annual cost of angina pectoris  Annual cost of OSA  CC costs 1st year  EC costs 1st year  BC costs 1st year  CC costs following years  EC cancer following years  BC costs following years | 220,90  2  5,254,34  5,508.42  7,990.21  10,386.38  9,587.43  5,297.82  3,920.82  22,941.47  23,438.81  24,481.97  4,813.54  2,920.33  1,027.12 | ±20%  1– 4  ±20%  ±20%  ±20%  ±20%  ±20%  ±20%  ±20%  ±20%  ±20%  ±20%  ±20%  ±20%  ±20% | GAMMA  GAMMA  GAMMA  GAMMA  GAMMA  GAMMA  GAMMA  GAMMA  GAMMA  GAMMA  GAMMA  GAMMA  GAMMA  GAMMA  GAMMA | Borisenko et al. 2017  Alakörkkö et al. 2023  Heisser et al. 2022  Kahn et al. 2023  Heisser et al. 2022  MW von EC und BC  Kahn et al 2023 |
| **Indirect cost inputs** | | | | |
| OR for sick leave days 25 - 29.9 kg/m2  OR for sick leave days 30 - 34.9 kg/m2  OR for sick leave days 35 -39.9 kg/m2  OR for sick leave days 40 - 44.9 kg/m2  OR for early retirement 25 - 29.9 kg/m2  OR for early retirement 30 - 34.9 kg/m2  OR for early retirement 35 -39.9 kg/m2  OR for early retirement 40 - 44.9 kg/m2  Average days per sick leave  Indirect costs per hour  Gross income per year  Early retirement rate  Employment rate  Sick leave days due to CC  Sick leave days due to EC  Sick leave days due to BC | 1.19  1.23  1.38  2.66  1.21  1.56  1.90  1.82  14.80  43.40  56,412.00  3.8%  78%  83.33  107.50  125.00 | 1.01-1.39  1.00-1.51  0.95-2.01  1.36-5.20  0.82-1.78  0.92-2.70  1.05-3.47  0.78-4.22  ±20%  ±20%  ±20%  ± 10%  ± 10%  ±20%  ±20%  ±20% | GAMMA  GAMMA  GAMMA  GAMMA  GAMMA  GAMMA  GAMMA  GAMMA  GAMMA  GAMMA  GAMMA  BETA  BETA  GAMMA  GAMMA  GAMMA | Yates et al. 2016  Destatis  Federal Ministry of Health |
| Discount rate costs | 3% | 0%-5% | BETA | IQWIG |

DHA: Digital health application; T2D: Type-2-Diabetes; ACS: Acute coronary syndrome; OSA: Obstructive sleep apnea; OR: Odds ratio; CC: Colon cancer; EC: Endometrial cancer; BC: Breast cancer; IQWIG: Institut für Qualität und Wirtschaftlichkeit im Gesundheitswesen

**References**

1. Alakörkkö I, Törmälehto S, Leppänen T, McNicholas WT, Arnardottir ES, Sund R. The economic cost of obstructive sleep apnea: A systematic review. Sleep Med Rev. 2023 Dec;72:101854. doi: 10.1016/j.smrv.2023.101854. Epub 2023 Sep 21. PMID: 37939650.
2. Borisenko O, Mann O, Duprée A. Cost-utility analysis of bariatric surgery compared with conventional medical management in Germany: a decision analytic modeling. BMC Surg. 2017 Aug 3;17(1):87. doi: 10.1186/s12893-017-0284-0. PMID: 28774333; PMCID: PMC5543597.
3. Heisser T, Simon A, Hapfelmeier J, Hoffmeister M, Brenner H. Treatment Costs of Colorectal Cancer by Sex and Age: Population-Based Study on Health Insurance Data from Germany. Cancers (Basel). 2022 Aug 8;14(15):3836. doi: 10.3390/cancers14153836. PMID: 35954499; PMCID: PMC9367511.
4. Institute for Quality and Efficiency in Health Care (IQWiG). General Methods. Version 8.0 [Internet]. Cologne 2025. <https://doi.org/10.60584/General-Methods_V8.0> [accessed 3. November 2026]
5. Khan SA, Hernandez-Villafuerte K, Hernandez D, Schlander M. Estimation of the stage-wise costs of breast cancer in Germany using a modeling approach. Front Public Health. 2023 Jan 6;10:946544. doi: 10.3389/fpubh.2022.946544. PMID: 36684975; PMCID: PMC9853539.
6. Yates N, Teuner CM, Hunger M, Holle R, Stark R, Laxy M, Hauner H, Peters A, Wolfenstetter SB. The Economic Burden of Obesity in Germany: Results from the Population-Based KORA Studies. Obes Facts. 2016;9(6):397-409. doi: 10.1159/000452248. Epub 2016 Dec 13. PMID: 27951530; PMCID: PMC5644792.
