# Supplementary material for: Long-Term Cost and Health Impact of a Digital Obesity Intervention in Germany: Economic Modeling Study
Source: JMIR Form Res. 2026 Aug 24;10:e88576. doi: 10.2196/88576 (PMC13550978; doi:10.2196/88576)
Supplement: Supplementary file 5 [file formative_v10i1e88576_app5.docx]

**Multimedia Appendix 5**

*Health state utilities*

Health-related quality of life was modeled using utility values obtained from published literature. Baseline utilities were age-dependent and adjusted by applying disutilities associated with elevated BMI and obesity-related comorbidities.

For each BMI category, a constant utility decrement was applied throughout the relevant period. Comorbidity-specific disutilities were incorporated as additive effects. Acute cardiovascular events such as myocardial infarction (MI) and stroke were modeled with event-specific disutility profiles: a larger utility loss was assigned during the year of the acute event, followed by a smaller, persistent decrement in subsequent years reflecting the long-term impact of post-MI or post-stroke status.

All disutilities were applied proportionally to the duration spent in the respective health state within each model cycle. In general, utility adjustments were implemented for the full length of each cycle. An exception was made for obstructive sleep apnoea (OSA), where diagnosis is typically delayed; thus, the associated disutility was applied only to half the annual cycle to reflect a mid-year onset assumption.

**Table S5.** Utility values.

| **Parameter** | **Base Case** | **Range** | **Distribution** | **Source** |
| --- | --- | --- | --- | --- |
| **Utilities** | | | | |
| Mean adult utility at age 35  Mean adult utility at age 45  Mean adult utility at age 49  Age (per year decrease) | 0,83  0,83  0,83  0,00 | ± 10%  ± 10%  ± 10%  ± 10% | BETA  BETA  BETA  BETA | Gulliford et al. 2016 |
| **Disutilities** | | | | |
| Disutility BMI 35 – 39  Disutility BMI ≥ 40  Disutility T2D  Disutility MI  Disutility Post MI  Disutility Stroke  Disutility Post Stroke  Disutility Angina pectoris  Disutility OSA  Disutility Cancer | 0,17  0,26  0,07  0,13  0,06  0,16  0,07  0,07  0,24  0,07 | ± 10%  ± 10%  ± 10%  ± 10%  ± 10%  ± 10%  ± 10%  ± 10%  ± 10%  ± 10% | BETA  BETA  BETA  BETA  BETA  BETA  BETA  BETA  BETA  BETA | Gulliford et al. 2023  Gulliford et al. 2023  Gulliford et al. 2023  Clarke et al. 2002  Assumption  Clarke et al. 2004  Kim et al 2022  Gulliford et al. 2022  Kim et al 2022  Kim et al 2023 |
| Discount rate utilities | 3% | 0%-5% | BETA | IQWIG |

IQWIG: Institut für Qualität und Wirtschaftlichkeit im Gesundheitswesen

**References**

1. Clarke P, Gray A, Holman R. Estimating utility values for health states of type 2 diabetic patients using the EQ-5D (UKPDS 62) Med Decis Making 2002;22:340–349.
2. Gulliford MC, Charlton J, Booth HP, Fildes A, Khan O, Reddy M, Ashworth M, Lit-tlejohns P, Prevost AT, Rudisill C. Costs and outcomes of increasing access to bariatric surgery for obesity: cohort study and cost-effectiveness analysis using electronic health records. Southampton (UK), 2016. https://www.ncbi.nlm.nih.gov/books/NBK362445/ doi: 10.3310/hsdr04170. Assessed 03.04. 2017
3. Institute for Quality and Efficiency in Health Care (IQWiG). General Methods. Version 8.0 [Internet]. Cologne 2025. <https://doi.org/10.60584/General-Methods_V8.0> [accessed 3. November 2026]
4. Kim N, Wang J, Burudpakdee C, Song Y, Ramasamy A, Xie Y, Sun R, Kumar N, Wu EQ, Sullivan SD. Cost-effectiveness analysis of semaglutide 2.4 mg for the treatment of adult patients with overweight and obesity in the United States. J Manag Care Spec Pharm. 2022 Jul;28(7):740-752. doi: 10.18553/jmcp.2022.28.7.740. PMID: 35737858; PMCID: PMC10372962.
