# Supplementary material for: Long-Term Cost and Health Impact of a Digital Obesity Intervention in Germany: Economic Modeling Study
Source: JMIR Form Res. 2026 Aug 24;10:e88576. doi: 10.2196/88576 (PMC13550978; doi:10.2196/88576)
Supplement: Supplementary file 6 [file formative_v10i1e88576_app6.docx]

M**ultimedia Appendix 6**

**Table S1.** Cost-effectiveness results (optimistic scenario: weight maintenance scenario).

| **Cost components** | **DiGA** | **care as usual** |
| --- | --- | --- |
| Oviva Direkt | 441.80 € |  |
| T2D costs | 9,132.12 € | 10,346.90 € |
| ACS | 148.44 € | 171.24 € |
| Post ACS | 829.26 € | 924.12 € |
| Stroke | 45.58 € | 45.62 € |
| Post Stroke | 351.02 € | 351.10 € |
| OSA | 2,827.57 € | 3,189.38 € |
| Cancer costs | 31.70 € | 31.99 € |
| **Total direct costs** | **13,807.47 €** | **15,060.35 €** |
| Sick leave costs (without cancer) | 43,043.04 € | 50,198.02 € |
| Early retirement costs | 9,148.36 € | 10,414.59 € |
| Sick leave days cancer | 171.72 € | 172.81 € |
| **Total indirect costs** | **52,363.12 €** | **60,785.42 €** |
| **TOTAL costs** | **66,170.59 €** | **75,845.77 €** |
| Cost difference | -9,675.18 € | |
| **QALYs** | 5.8917 | 5.6883 |
| QALY difference | 0.2034 | |
| **ICUR /QALY** | **DOMINANT (-47,557.34 €)** | |
| **NMB** | 20,220.20 € | |
| LYs | 8.3481 | 8.3438 |
| LY difference | 0.0043 | |
| **ICER /QALY** | **DOMINANT (-2,250,976.12 €)** | |

DiGA: Digital health application; T2D: Type-2-Diabetes; ACS: Acute coronary syndrome; OSA: Obstructive sleep apnea; QALY: Quality-adjusted life-year; LY: life year; ICUR: incremental cost utility ratio; NMB: Net Monetary benefit; ICER: incremental cost effectiveness ratio

**Table S2.** Cost-effectiveness results (pessimistic scenario: 100% weight regain scenario).

| **Cost components** | **DiGA** | **care as usual** |
| --- | --- | --- |
| Oviva Direkt | 441.80 € |  |
| T2D costs | 10,029.74 € | 10,503.85 € |
| ACS | 152.87 € | 171.44 € |
| Post ACS | 849.20 € | 925.06 € |
| Stroke | 46.00 € | 45.90 € |
| Post Stroke | 353.82 € | 352.95 € |
| OSA | 3,318.74 € | 3,372.57 € |
| Cancer costs | 31.77 € | 31.99 € |
| **Total direct costs** | **15,223.931 €** | **15,403.76 €** |
| Sick leave costs (without cancer) | 51,084.15 € | 51,322.81 € |
| Early retirement costs | 10,154.32 € | 10,558.28 € |
| Sick leave days cancer | 172.02 € | 172.84 € |
| **Total indirect costs** | 61,634.41 € | 62,053.93 € |
| **TOTAL costs** | **76,634.41 €** | **77,457.69 €** |
| Cost difference | -823.28 € | |
| **QALYs** | 5.6341 | 5.6294 |
| QALY difference | 0.0047 | |
| **ICUR /QALY** | **DOMINANT (-174,188.22 €)** | |
| **NMB** | **1,083.18 €** | |
| **LYs** | 8.3419 | 8.3411 |
| LY difference | 0.0008 | |
| **ICER /QALY** | **DOMINANT (-999,092.22 €)** | |

DiGA: Digital health application; T2D: Type-2-Diabetes; ACS: Acute coronary syndrome; OSA: Obstructive sleep apnea; QALY: Quality-adjusted life-year; LY: life year; ICUR: incremental cost utility ratio; NMB: Net Monetary benefit; ICER: incremental cost effectiveness ratio
