# Supplementary material for: Long-Term Cost and Health Impact of a Digital Obesity Intervention in Germany: Economic Modeling Study
Source: JMIR Form Res. 2026 Aug 24;10:e88576. doi: 10.2196/88576 (PMC13550978; doi:10.2196/88576)
Supplement: Supplementary file 7 [file formative_v10i1e88576_app7.docx]

**Multimedia Appendix 7**

**Figure S1. Deterministic one-way sensitivity analysis from the payer perspective** visualized as Tornado plots. Deterministic sensitivity analysis was used to identify the critical variables affecting risk analysis. Results are displayed as Tornado diagrams for the 30 variations with the greatest impact, where each bar represents a one-way sensitivity analysis, and width of bars represents impact on model results. The ICUR is plotted on the x-axis.

(a) **Cost-effectiveness plane**

(b) **Cost-effectiveness acceptability curves**

**Figure S2.** **Probabilistic sensitivity analysis from the payer perspective**: (a) Cost-effectiveness plan showing the results of the Monte Carlo probabilistic sensitivity analysis for 1,000 patients. Incremental cost is plotted on the Y-axis, incremental effectiveness is plotted on the X-axis. (b) Cost-effectiveness acceptability curves display the percentage of iterations that favor the use of a digital health application in comparison to care as usual, over a range of willingness-to-pay. The X-axis displays reported values as € per QALY.
